# The Incidence Proportion of SARS-CoV-2 Infections and the Percentage of Deaths among Infected Healthcare Workers in Poland

**DOI:** 10.3390/jcm12113714

**Published:** 2023-05-27

**Authors:** Marek Wojczyk, Ewa Niewiadomska, Małgorzata Kowalska

**Affiliations:** 1 Doctoral School, Medical University of Silesia, 40-055 Katowice, Poland; 2 Department of Epidemiology and Biostatistics, Faculty of Health Science, Medical University of Silesia, 41-902 Bytom, Poland; 3 Department of Epidemiology, Faculty of Medical Science, Medical University of Silesia, 40-055 Katowice, Poland

**Keywords:** COVID-19, healthcare workers, pandemics, SARS-CoV-2, vaccination coverage

## Abstract

(1) Background: The incidence proportion of SARS-CoV-2 infection among healthcare workers (HCWs) in Poland is not exactly known. This study aims to present secondary epidemiological data identifying the scale of the spread of novel coronavirus infection and the estimation of vaccination coverage in selected professional groups of HCWs in Poland. (2) Methods: The secondary epidemiological data included both the number of infections and infection fatality rate (IFR) in individual occupational groups, which occurred throughout the observation period (January 2021–July 2022), both in the country and in individual voivodeship (administrative area). (3) Results: The incidence proportion of SARS-CoV-2 infections among HCWs was 16.48%. The highest percentage of infected workers concerned laboratory scientists (21.62%) and paramedics (18%). The highest frequency of infections among HCWs occurred in the province of Zachodnio-Pomorskie (18.9%). Due to COVID-19, 558 healthcare workers died during the analysed period, mostly nurses (*n* = 236) and doctors (*n* = 200). The results regarding the vaccination coverage of HCWs against COVID-19 indicate the highest percentage of vaccinated were among doctors (83.63%) and the lowest among physiotherapists (38.2%). (4) Conclusions: In general, the percentage of infections was high in Poland during the pandemic (16.48%). Significant territorial differences were observed in the frequency of infections, deaths, and percentage of vaccinated workers in individual voivodeships.

## 1. Introduction

The incidence proportion of SARS-CoV-2 infection among healthcare workers (HCWs) in Poland is not exactly known. So far, attempts to determine the number of infected HCWs have been limited to estimating the incidence based on cross-sectional studies, often single-centre [1,2,3]. Naturally, the infection rate was related to the period of observation in a particular phase of the epidemic. According to data from other centres, the average incidence was 8.5% in the initial phase of the pandemic in Europe (2020) [4], and in Poland, the value determined based on a multi-centre study was 25.2% in 2021 [5].

In February 2022, the Ministry of Health announced that the number of infected medics since the beginning of the pandemic was over 200,000 [6]. Despite the data provided by the Ministry on the number of infections among HCWs since September 2020 [7], a separate register enabling the analysis of the necessary epidemiological data in individual professional groups was implemented in Poland only in January 2021. Earlier information on the scale of infections among HCWs was incomplete and concerned only with selected medical professions [8,9]. Data collected from 1 January 2021 allows for more accurate tracking of both the numbers of infected and deceased medical workers, as well as the percentage of fully vaccinated HCWs in our country, depending on the region.

Knowing the incidence proportion of novel coronavirus infections among HCWs has a huge impact on maintaining the quality of healthcare during the epidemiological crisis that the COVID-19 pandemic has become. Infections of people working in the health care system directly affect, among others, absenteeism at work (nationally, an increase from 5.6% in 2019 to 9.3% in 2020) [10], which causes an increase in the workload of staff performing their professional duties, results in longer waiting times for scheduled diagnostic appointments and treatment procedures, and worsens the quality of care provided to patients, also for COVID-19 [10,11,12].

This study aimed to present secondary epidemiological data identifying the scale of the spread of novel coronavirus infection in selected professional groups of HCWs in Poland. In addition, the study provides data about the infection fatality rate (IFR) among selected medical professions and their vaccination status against COVID-19.

## 2. Materials and Methods

Secondary epidemiological data provided by the e-Health Centre, a unit subordinate to the Ministry of Health, was obtained for the study [13]. The data included both the number of SARS-CoV-2 infections registered among HCWs and the number of deaths, as well as the number of doses of COVID-19 vaccinations. Confirmation of infection was carried out by both PCR and antigen tests in facilities conducting identification of infections throughout the country. All of the positive results were reported to the database of infected persons practicing the medical profession. However, data available in the registry do not allow for recognition of the number of cases confirmed with the use of particular individual testing methods.

The data obtained included the employee’s occupational group, place of employment in the individual voivodeships (administrative area), and the month in which the infection or death occurred. In addition, the number of vaccinations performed in individual occupational groups in all voivodeships was analysed. The observation period covered the time from January 2021 to July 2022. The data embraced particular categories of medical professions, including the following: doctor, nurse, paramedic, laboratory scientist, dentist, pharmacist, physiotherapist, and midwife.

After organizing the collected data, the numbers of first-time and all infections as well as deaths in individual occupational groups that occurred during the observation period, both in the whole country and in individual voivodeships, were summed. Then, taking into account the data identifying the number of medical employees authorized to practice [14], the percentages of first-time infected staff and all other employees of selected occupational groups (doctors, dentists, nurses, midwives, laboratory scientists, and pharmacists) were calculated. Concerning the group of paramedics, the source data only present the number of people working in the State Medical Rescue System, so in the absence of more precise data, this number was adopted as the official number of paramedics employed in our country (as a denominator). Similarly, for the group of physiotherapists, we only had access to the number of physiotherapists employed in the public health care system [14]. Considering the large number of specialists in this professional category working outside public health care units, it was decided that the data of the National Chamber of Physiotherapy should be used to determine the percentage of infections in this group (as a denominator) [15].

To determine the percentage of deceased employees in particular occupational groups, the authors decided to present the IFR (infection fatality rate) instead of the fatality rates due to the low number of deaths in individual HCW groups. The infection fatality rate (IFR) was calculated by determining the percentage of deaths in a group of infected people for a specific occupational group [16].

At the same time, data were obtained on the number of vaccinations against COVID-19 in individual professional groups in all voivodeships in subsequent months, starting from December 2020 (the beginning of the vaccination campaign) until July 2022. Although these data identify the number of vaccination doses, there is no information on how many employees were vaccinated. In addition, accounting for the considerable dynamics of changes in the availability of subsequent vaccines and a different vaccination schedule, including the introduction of a single-dose vaccine in April 2021 [17] and the possibility of performing another (booster) vaccination from September 2021, the following procedure was adopted to calculate the percentage of staff vaccination status. The number of vaccination doses performed, reported from December 2020 to April 2021, was divided by two due to the availability of two doses of preparations for one employee in the period under review. It was assumed that this is the number appropriate for determining the percentage of vaccinated employees in particular occupational categories and entitled to receive vaccination by the rules in force at the time [18].

Due to the scope of available data (cumulative number of vaccinations performed, without considering the dose number or the name of the preparation), the authors refrained from attempting to interpret the vaccination status of individual professional groups in terms of booster doses.

Descriptive statistics methods were used in the data analysis because the project was a descriptive epidemiological study. The number and percentages of staff infected with SARS-CoV-2, the IFR value, and the percentage of those vaccinated with two doses in the individual voivodships, with the separation of the professional category, are presented. The secondary nature of the obtained registry data made impossible a comparison of the expected groups. According to the accepted epidemiology methods dedicated to the descriptive trials [19], the authors were able only to recognize temporal or spatial variability without the possibility to assess the relationship between exposure and health outcomes (lack of data about exposure or individual determinants). The capabilities of Microsoft Office Excel 4.0 software were used, and QGIS 3.16 was used for map construction, license: GNU GENERAL PUBLIC LICENSE, Copyright (C) 1989, 1991 Free Software Foundation, Inc.

## 3. Results

During the analysed period, from 1 January 2021 to 30 June 2022, a total of 114,213 SARS-CoV-2 infections were registered in Poland among HCWs, which accounted for 16.48% of all employees authorized to practice during that time. It is worth noting that the vast majority of infections were first-time infections (*n* = 98,896; 86.59% of all notifications).

**Figure 1:**
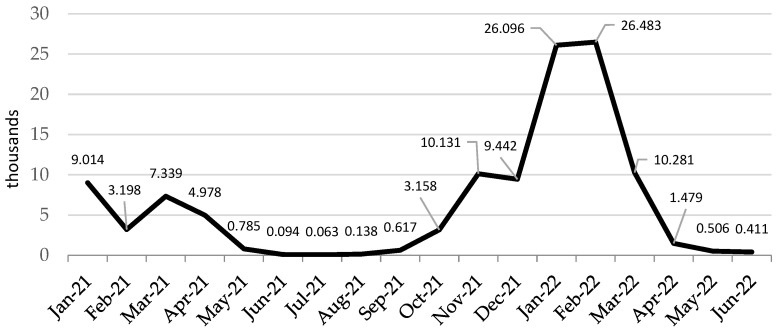
Dynamics of changes in the total number of infected healthcare workers in individual months of the study period (1 January 2021–30 June 2022) in Poland.

Similarly to the general population, the occurrence of an increased number of infections among HCWs was influenced by the period of increased virus infectivity. In each phase of the epidemic, we were dealing with a slightly different predominance of mutations and subsequent variants of the SARS-CoV-2 responsible for most infections: by June 2021—alpha variant, by January 2022—delta variant, by the end of February 2022 omicron BA.1, and by the end of June 2022—Omicron BA.4 [20]. Representatives of medical professional groups were infected most frequently in January and February 2022. More than 25,000 HCWs became infected in these two months (Figure 1).

The percentage of infected HCWs in particular professional categories varied, with the highest among laboratory scientists (21.62%), paramedics (18.34%), and nurses (18.07%). The lowest percentage of HCWs infected with coronavirus was registered by dentists (8.62%). The full list of values describing the percentage of infected HCWs in the individual professional categories by voivodeships is presented in Table 1.

**Figure 2:**
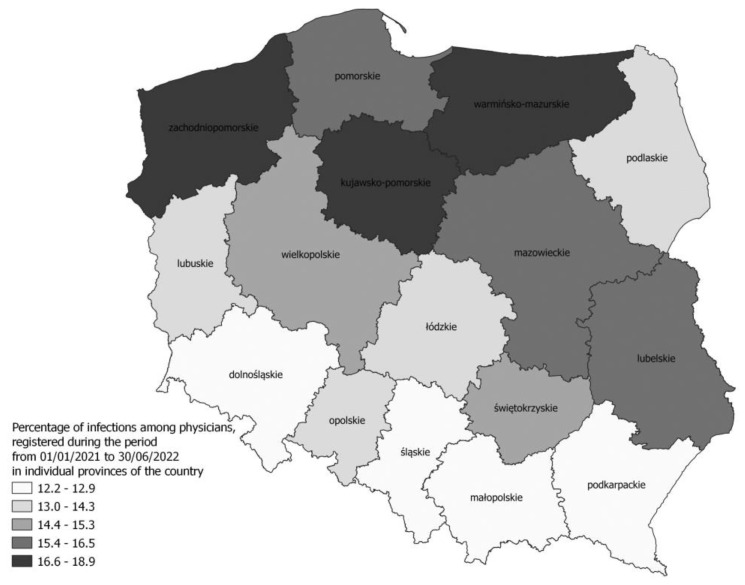
Percentage of infections among physicians, registered during the period from 1 January 2021 to 30 June 2022 in individual provinces of the country.

Figure 2 shows a sample map identifying regional differences in the percentage of infected physicians. The highest values were recorded in the voivodeships: Zachodniopomorskie (18.9%), Warmińsko-Mazurskie (18.2%), and Kujawsko-Pomorskie (18.5%). The lowest percentages of infected doctors were observed in the voivodeships of Podkarpackie (12.2%), Dolnośląskie (12.92%), Małopolskie (12.96%), and Śląskie (13.0%). The highest number of recurrent SARS-CoV-2 infections concerned HCWs from the Kujawsko-Pomorskie voivodeship, while the lowest percentage of recurrent infections was recorded in Małopolska.

**Figure 3:**
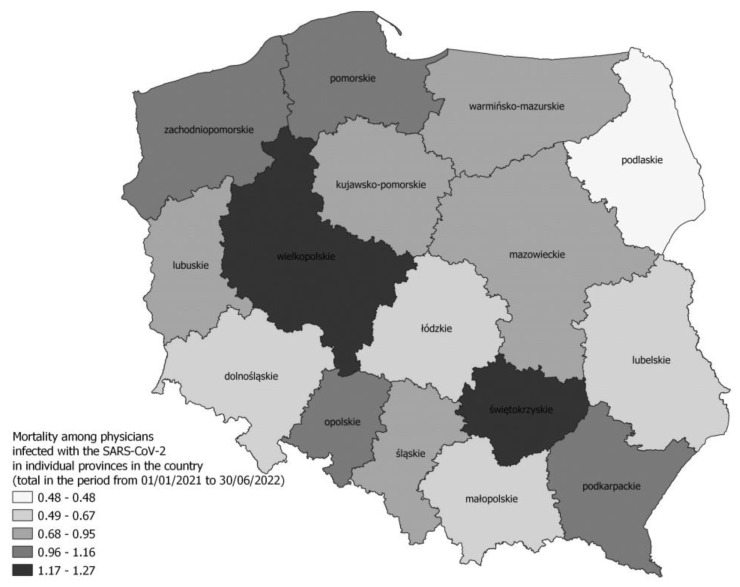
Mortality among physicians infected with the SARS-CoV-2 in individual provinces in the country (total in the period from 1 January 2021 to 30 June 2022).

Due to COVID-19, 558 HCWs died during the analysed period, of which the largest groups were nurses (*n* = 236) and doctors (*n* = 200). However, the highest infection fatality rate (IFR) concerned the group of dentists (1.45%). The lowest IFR value concerned physiotherapists (0.06%). Detailed data on the differences in the IFR depending on the professional group and voivodship are presented in Table 2. Figure 3, in turn, presents a map of the percentage of registered deaths among infected physicians.

**Figure 4:**
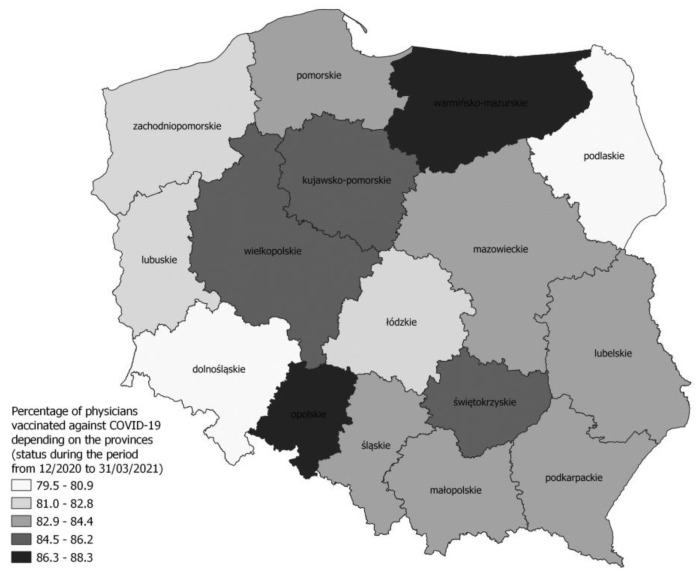
Percentage of physicians vaccinated against COVID-19 depending on the provinces (status during the period from December 2020 to 31 March 2021).

The results regarding the vaccination status of HCWs against COVID-19 indicate that during the period from December 2020 to March 2021, medical doctors were the group with the highest percentage of vaccination (83.63%) and the lowest were physiotherapists (38.2%). Detailed data in this regard, including voivodeships, is presented in Table 3. In turn, Figure 4 presents regional differences in the percentage of vaccinated doctors.

## 4. Discussion

The results of the presented study revealed significant variability in the number of HCWs infected by SARS-CoV-2 in particular occupational groups as well as observed spatial variability. The highest infection proportion was identified in the following professions: laboratory scientists, paramedics, and nurses. In the group of infected workers, the highest percentage of deaths was recorded in dentists. Moreover, the level of vaccination of medical workers varied, with the highest percentage in physicians, and the lowest in physiotherapists. Unfortunately, the secondary nature of the obtained data does not allow for an unambiguous assessment of the impact of vaccination on the IFR value.

In September 2021, the World Health Organization estimated that, by May 2021, between 80,000 and 180,000 HCWs had died of COVID-19 [21]. At the same time, it was possible to determine, based on data from 119 countries, that by September 2021, about 40% of HCWs had been vaccinated against the disease [21]. However, due to the lack of accurate reports and the reluctance of some countries to publish accurate summaries [4], it is difficult to verify the above estimates. A local, multi-centre study conducted in Poland indicated that the percentage of infections in the group of HCWs was 25.2% [5]. At the same time, a seroepidemiological study conducted in the Upper Silesian agglomeration in the first phase of the epidemic (until the end of 2021) indicated that the probable infection rate of HCWs was in the range of 16.1–22.5% [3]. The results collected from the officially functioning register and presented in this paper show significant differences concerning the data from other research centres. In general, it can be seen that the percentage of infected HCWs in other countries did not significantly exceed 10% [22]. However, the cited data have most often been derived from cross-sectional studies, and the percentages of infections given in them are the result of estimations. The approximate similar percentage of SARS-CoV-2 infections among HCWs to that calculated by us in the presented work was revealed in a study from Malaysia in 2021. The percentage of infected HCWs was 17.4% in their research, which was similar to our study; at the same time paramedics were most often infected as well (68.4%) [23].

The risk of SARS-CoV-2 infection in HCWs was higher than the risk observed in the general population, but it is worth noting that it varied within medical and healthcare professions and the exact workplace, including the nature of the ward [24,25]. An analysis of data on medical personnel in Canada indicated that people employed in healthcare facilities accounted for 7% of all infected. At the same time, women and younger people employed in healthcare were more likely to suffer from COVID-19 than in the general population, and the risk of hospitalization and death was lower in this group than in the general population [26].

The results of another international study confirmed that declarations of HCWs regarding sufficient preparation for work in a pandemic more often concerned nurses than physicians, and at the same time more often men than women [27]. Despite the satisfactory results of this self-assessment, nurses in Poland were infected more often than medical doctors, which was reflected in the data presented in our study. Probably, the different nature of work with patients, as well as the frequency and length of contact with patients, are important factors for the observed variation in the percentage of coronavirus infections among HCWs [22,25]. The longer procedures and the nature of the care specific for paramedics or nurses mean that the percentage of infected in these groups observed in the registers was higher than, for example, recorded in medical doctors. It is worth emphasizing here that the high frequency of SARS-CoV-2 infections in the groups of paramedics and nurses does not correspond to the highest percentage of deaths in the infected, respectively IFR = 0.11% for paramedics and IFR = 0.42% in the group of nurses. The highest percentage of deaths among infected employees concerned dentists (IFR = 1.45%) and doctors (IFR = 0.85%). This is a hard-to-explain observation based on secondary epidemiological data and a descriptive type of study. One of the possible hypotheses to explain this is based on the relatively high percentage of older age (over 65) current physicians in Poland. Similar conclusions were drawn during the analysis of international data from the beginning of the COVID-19 pandemic, where more frequent deaths were recorded among male doctors than other HCWs, reflecting their advanced age [25]. It is obvious that the risk of chronic diseases increases with age [28] and comorbidity is very common [29]. According to the data available from the Central Statistical Office, although the average age of doctors and nurses was the same (49.2 years; dentist—46 years; laboratory scientists—45 years), in the group of doctors, 28% of all doctors working in direct contact with patients were over 60 years of age. Dentists in this age group accounted for 17.7% and nurses for 16.3%. Moreover, 1.4% doctors and 0.4% dentists were over 80 [30]. For the other professions, there are no equivalent precise data.

It is very difficult to explain why an observed lower value of IFR was accompanied by a higher level of infected workers. The phenomenon of the highest percentage of infections occurring in the group of laboratory scientists could be explained by the observation that, during the pandemic, these workers also participated in biological sampling procedures from infected patients, as more nurses were needed to be focused on duties in hospitals rather than centres of testing. On the other hand, the younger age of laboratory scientists compared to physicians and nurses could be a key to a potential explanation. However, this explanation is only a potential hypothesis demanding future confirmation. As demonstrated in previous publications, the risk of death in patients with COVID-19 increases with comorbidity [31,32]. In our study, the average IFR value for all analysed professional groups was 0.48%, which is important; the average mortality rate reported in HCWs from 37 countries was at a similar level, i.e., 0.05 per 100,000 [33]. Unfortunately, the lack of relevant data in the register identifying the age of individual HCWs or their current health status (information on diagnosed diseases) made it impossible to unequivocally assess the issue raised.

The level of vaccination against COVID-19 in the group of HCWs in Poland during the initial period of vaccination availability was not very high and amounted to 61.48% (detailed data are presented in Table 3). Such a low level of vaccination was not due to the lack of availability of the vaccine but was rather the result of employees’ limited trust in the preparations used for vaccination against COVID-19 [34]. The greatest objections were declared by nurses, in the Linder-Pawłowicz study, 64.7% of surveyed physicians and 63.7% of medical students, and only 34.5% of nurses indicated trust in vaccination, while 46.6% of nurses and 40% of pharmacists did not have trust [35]. In our study, the lowest vaccination status against COVID-19 during the initial phase of vaccination availability was observed in the group of physiotherapists (38.2%). This is an important observation, especially since the results of a seroepidemiological study in Great Britain confirmed that the greatest risk of SARS-CoV-2 infection concerned physiotherapists (OR 2.78; 95% CI: 1.21–6.36) [36]. Their professional contact most often involves the care of elderly patients, often with multiple comorbidities, so it is worth including these issues in the training programs of future specialists. A low percentage of vaccinated employees in our study also concerned nurses (54.5%) and midwives (54.4%). The highest percentage of vaccinated HCWs was recorded in the group of medical doctors (83.63%), although some territorial variability of the indicator in individual voivodeships was noticed. The worst situation in this respect was in the Podlaskie voivodeship (79.47%). It is worth adding here that also in other countries worldwide, physicians are the group of HCWs that most favours the obligation to vaccinate against COVID-19 [24,36,37].

In conclusion, it should be noted that in comparison to data from other research centres in various countries, the incidence proportion of SARS-CoV-2 infection in Poland was quite high. As for the COVID-19 vaccination prophylaxis, it was confirmed that the percentage of vaccinated HCWs differed in individual occupations, with doctors among the most frequently vaccinated.

A certain limitation of the study was the secondary nature of the analysed data and their scope. The data relate to a period of 18 months of the pandemic, and their arbitrary, rather simplified nature does not allow for conclusions about infection risk factors apart from variables related to the profession and place of work (voivodeship). Moreover, it was difficult to indicate the exact percentage of infected individuals in selected professional groups because there is no precise register of the number of persons authorized and performing particular medical professions in our country, hence the calculated values may be underestimated from the actual percentage of infected persons.

Unfortunately, as the article concerns secondary epidemiological data (available registry), we were unable to verify the potential underestimation. However, current published data suggest that the proportion of asymptomatic infection caused by Omicron was calculated as 25.5% (95% CI: 17.0–38.2%) and nonsevere illness as 97.9% (95% CI: 97.1–98.7%) comparing to Delta variant with 8.4% (95% CI: 4.4–16.2%) asymptomatic and 91.4% (95% CI: 87.0–96.0%) nonsevere [38]. Other authors have calculated the pooled percentage of asymptomatic infections of the Omicron variant as 32.40% (95% CI: 25.30–39.51%) [39]. Moreover, a Malesian multicentre cross-sectional study proves that the 50.9% total prevalence of SARS-CoV-2 infections among HCWs in the period between March 2020 and April 2022 for up to 80.1% was due to the Omicron variant of the virus [40]. Similarly, the determination of the percentage of the vaccinated medical workers has some limitations. Due to the lack of precise data on the number of the vaccinated HCWs, the percentage of the vaccinated was estimated based on the adopted methodology (the number of doses administered in individual occupational groups divided by two corresponded to the estimated number of the vaccinated) for the selected period when two-dose preparations were available. However, it was recognized that the presented results may be a form of supplement to the gap resulting from the lack of more reliable data in Poland.

## 5. Conclusions

The percentage of SARS-CoV-2 infections among HCWs in general during the pandemic in Poland was high and amounted to 16.48%. Laboratory scientists, paramedics, and nurses were the most often infected groups of medical staff. The highest percentage of deaths was recorded in the professional group of dentists. Physicians accounted for the largest percentage of medical workers vaccinated against COVID-19. Significant territorial differences were observed in the frequency of infections, the percentage of deaths, and the percentage of vaccinated HCWs in individual voivodeships. However, the accurate number of infected HCWs was difficult to estimate due to the limitations in the nature of the presented data as well as the dynamics of the epidemiological situation. Similar to the general population, the spread of new variants of the SARS-CoV-2 virus with lower impact on the number of patients in a critical condition caused the number of HCWs who experienced SARS-CoV-2 to be underestimated.

## Figures and Tables

**Table 1:** Percentage of SARS-CoV-2 infections among HCWs (in total and first-time) in the individual voivodship (1 January 2021–30 June 2022).

Voivodship	Infections	%DOC	%NUR	%PM	%DEN	%LAB	%PHA	%PHY	%MID
Total in country	Generally	14.99	18.07	18.34	8.62	21.62	14.44	16.92	16.84
	First-time	13.12	15.18	14.91	8.19	19.40	13.56	15.11	15.05
Dolnośląskie	Generally	12.92	17.26	16.20	6.59	21.27	13.82	15.98	16.65
	First-time	11.50	14.79	12.64	6.13	19.04	13.10	14.56	14.97
Kujawsko-pomorskie	Generally	18.55	23.25	16.12	12.47	26.00	20.41	19.37	18.74
	First-time	16.19	18.95	12.00	12.06	23.07	18.05	16.70	15.85
Łódzkie	Generally	13.99	17.14	24.15	8.90	19.41	14.14	15.58	16.42
	First-time	12.05	14.17	17.53	8.40	18.07	13.22	14.10	14.55
Lubelskie	Generally	16.30	22.13	19.38	9.78	23.32	15.53	16.26	20.49
	First-time	13.58	18.06	16.83	9.58	20.85	14.54	16.26	17.51
Lubuskie	Generally	14.14	18.55	20.95	6.65	18.43	13.98	22.02	16.93
	First-time	12.69	15.74	17.06	6.32	16.08	13.40	13.68	15.45
Małopolskie	Generally	12.96	15.51	13.95	7.88	17.67	13.85	14.13	14.16
	First-time	12.04	13.03	12.50	7.43	16.34	13.27	12.81	13.01
Mazowieckie	Generally	16.25	18.35	23.21	9.46	22.34	16.29	18.29	16.99
	First-time	14.43	15.84	19.12	9.14	20.41	15.37	16.55	15.51
Opolskie	Generally	13.79	17.96	16.46	7.76	22.18	16.72	14.97	19.22
	First-time	12.23	15.15	14.71	7.51	17.41	14.81	13.35	16.90
Podkarpackie	Generally	12.25	16.04	12.13	6.03	23.16	10.65	14.60	13.81
	First-time	10.14	13.36	10.50	5.73	17.34	10.29	12.95	12.62
Podlaskie	Generally	14.39	17.06	17.03	8.60	18.84	10.71	15.51	15.30
	First-time	12.41	14.54	12.30	8.10	16.60	10.37	13.62	14.02
Pomorskie	Generally	16.60	19.26	13.59	10.61	24.13	16.04	20.52	17.30
	First-time	14.70	16.45	10.73	10.07	22.07	14.75	18.18	15.64
Śląskie	Generally	13.00	17.00	17.62	7.28	20.51	12.91	17.92	15.85
	First-time	11.40	14.44	14.76	6.97	18.34	12.11	16.10	14.36
Świętokrzyskie	Generally	15.10	15.03	16.93	6.02	16.44	8.81	13.49	13.86
	First-time	12.69	12.75	15.34	5.78	15.69	8.59	12.12	13.39
Warmińsko-Mazurskie	Generally	18.23	21.42	24.16	9.04	21.47	16.10	19.82	20.77
	First-time	14.91	17.16	19.52	8.22	18.76	15.53	16.56	18.30
Wielkopolskie	Generally	15.40	16.83	23.64	8.97	23.05	13.54	16.73	17.52
	First-time	13.46	14.09	18.47	8.40	21.27	12.85	14.99	15.30
Zachodnio-pomorskie	Generally	18.86	21.55	21.25	10.70	25.33	14.43	19.67	20.92
	First-time	16.40	17.53	17.46	9.88	23.22	13.36	17.36	17.86

Legend: DEN, dentists; DOC, medical doctors; HCWs, healthcare workers; LAB, laboratory scientists; MID, midwives; NUR, nurses; PHA, pharmacists; PHY, physiotherapists; PM, paramedics.

**Table 2:** Percentage of deaths among HCWs infected with the SARS-CoV-2, taking into account the voivodship and professional category.

Voivodship	%DOC	%NUR	% PM	% DEN	% LAB	% PHA	% PHY	% MID
Total in country	0.85	0.42	0.11	1.45	0.13	0.43	0.06	0.42
Dolnośląskie	0.59	0.50	0.48	1.57	0.00	0.47	0.10	0.00
Kujawsko-Pomorskie	0.87	0.43	0.00	3.23	0.34	0.61	0.00	0.77
Łódzkie	0.64	0.62	0.00	0.98	0.00	0.24	0.00	0.43
Lubelskie	0.67	0.43	0.55	2.05	0.35	0.61	0.15	0.00
Lubuskie	0.79	0.55	0.00	0.00	0.00	0.00	0.00	0.67
Małopolskie	0.60	0.19	0.00	0.64	0.00	0.42	0.09	0.81
Mazowieckie	0.77	0.33	0.00	1.24	0.17	0.10	0.05	0.55
Opolskie	1.10	0.64	0.00	4.92	0.00	0.00	0.00	0.55
Podkarpackie	1.13	0.51	0.00	0.83	0.00	0.68	0.00	0.51
Podlaskie	0.48	0.45	0.00	0.73	0.00	0.00	0.00	1.32
Pomorskie	1.06	0.16	0.00	1.58	0.00	0.46	0.00	0.27
Śląskie	0.94	0.55	0.00	2.00	0.26	0.67	0.07	0.39
Świętokrzyskie	1.27	0.51	0.00	1.37	0.00	2.50	0.24	0.48
Warmińsko-Mazurskie	0.95	0.31	0.58	3.03	0.90	0.88	0.25	0.41
Wielkopolskie	1.19	0.52	0.00	1.22	0.00	0.41	0.00	0.28
Zachodniopomorskie	1.16	0.32	0.00	0.45	0.00	0.50	0.00	0.00

Legend: DEN, dentists; DOC, medical doctors; HCWs, healthcare workers; LAB, laboratory scientists; MID, midwives; NUR, nurses; PHA, pharmacists; PHY, physiotherapists; PM, paramedics.

**Table 3:** Percentage of HCWs vaccinated against COVID-19 during the period from December 2020 to 31 March 2021, data by voivodship.

Voivodship	% DOC	% NUR	% PM	% DEN	% LAB	% PHA	% PHY	% MID
Total	83.63	54.53	63.14	70.85	65.70	61.35	38.20	54.43
Dolnośląskie	80.92	54.63	61.28	69.87	71.07	60.68	38.97	55.49
Kujawsko-Pomorskie	86.16	62.85	51.55	76.51	66.59	74.42	48.78	61.08
Łódzkie	82.11	56.84	67.59	67.87	61.68	57.07	39.81	53.41
Lubelskie	83.48	52.98	60.12	71.53	59.77	73.20	32.47	51.70
Lubuskie	82.81	58.84	71.60	71.54	67.25	72.98	39.81	56.25
Małopolskie	83.65	53.11	63.72	71.55	65.08	57.25	35.18	51.99
Mazowieckie	83.53	54.74	83.27	68.23	68.10	59.15	42.08	58.43
Opolskie	87.39	55.46	60.72	77.16	62.80	77.61	36.40	50.00
Podkarpackie	83.49	43.68	42.26	68.60	53.09	66.45	28.63	46.72
Podlaskie	79.47	47.35	45.74	65.60	54.61	58.58	28.79	50.17
Pomorskie	83.48	60.38	51.83	70.31	72.35	58.51	47.64	60.78
Śląskie	84.38	49.52	62.67	72.41	65.10	56.81	36.90	48.81
Świętokrzyskie	85.95	55.29	71.31	71.37	59.43	67.79	35.84	51.40
Warmińsko-Mazurskie	88.26	62.78	72.96	76.89	72.15	80.13	41.11	65.19
Wielkopolskie	85.62	59.08	77.94	74.79	72.36	61.85	38.42	57.32
Zachodniopomorskie	82.45	57.95	68.32	71.14	70.05	49.54	38.99	57.84

Legend: DEN, dentists; DOC, medical doctors; HCWs, healthcare workers; LAB, laboratory scientists; MID, midwives; NUR, nurses; PHA, pharmacists; PHY, physiotherapists; PM, paramedics.

## Data Availability

Data available on request. The data presented in this study are available on request from the corresponding author. The data are not publicly available as they were received from the “e-Health Centre” database in response to personal request.
